# OP04 The Modernized Cardiff Model: Multifaceted Modeling In The Era Of Cardiovascular-Kidney-Metabolic Syndrome

**DOI:** 10.1017/S0266462324000680

**Published:** 2025-01-07

**Authors:** Philip McEwan, Volker Foos, Jieling Chen, Marc Evans, Geraint Roberts, Robert Jenkins, Andrew Gibson

## Abstract

**Introduction:**

In the era of cardiovascular-kidney-metabolic syndrome, thorough evaluation of medicines with multiple treatment effects/indications demands a multifaceted modeling philosophy, despite the requirement of health technology assessment (HTA) models to focus on one disease. Using Cardiff, a model previously built for type 2 diabetes (T2D), we illustrate the changes needed to capture contemporary, holistic, patient-centered decision-making, and argue that HTA bodies should revise their approach.

**Methods:**

The upgraded model enables therapy selection and escalation determined by HbA1c thresholds, cardiovascular risk (QRISK3), comorbidities (established cardiovascular or chronic kidney disease), and weight (body mass index ≥35 kg/m^2^). Risk factor trajectories were updated by incorporating UKPDS-90 equations and other relevant data sources. Clinical outcomes were predicted using new risk equations incorporating cardiovascular outcomes trial data whenever possible. The updated model was applied to assess quality-adjusted life years (QALYs) and lifetime costs in newly diagnosed T2D patients in the UK, modeled via a conventional glycemic-centric approach versus a multifactorial treatment algorithm. Extrapolation to the national level utilized estimates of annual incidence.

**Results:**

The updated treatment algorithm captured and quantified the impact of nuanced comorbidity management called for in guidelines. In a cohort of newly diagnosed T2D patients, 81 percent initiated an SGLT2 inhibitor within five years, predominantly due to increasing cardiovascular risk, versus zero percent when escalation was dictated by HbA1c alone. Broad, early use of SGLT2 inhibitors resulted in an additional 0.73 predicted QALYs and GBP10,757 (USD13,600) in predicted lifetime cost savings per patient versus a “traditional” approach. Cost savings were primarily due to avoided renal events; extrapolation to the national level predicted cost savings to the payer of GBP2.8 billion (USD3.5 billion), which traditional models cannot capture.

**Conclusions:**

The modernized Cardiff model incorporates multifactorial prescribing guidelines and contemporary evidence around cardio-renal protection and is more adept at modeling costs and outcomes of multidimensional antidiabetic treatments; traditional glucose-centric modeling methods may introduce bias. Economic modeling and HTA processes must adapt to follow the complexities of modern disease management and remain relevant as healthcare systems address the cardiovascular-kidney-metabolic syndrome epidemic.

